# Psychological Drivers of Intentions Not to Waste Food: Evidence from an Extended Theory of Planned Behavior Model

**DOI:** 10.3390/foods15071194

**Published:** 2026-04-02

**Authors:** Hyosun Jung, Yu Hyun Hwang, Hye Hyun Yoon

**Affiliations:** 1 Center for Converging Humanities, Kyung Hee University, Seoul 02447, Republic of Korea; 2 College of Hotel & Tourism Management, Kyung Hee University, Seoul 02447, Republic of Korea; yuhyunhwang@naver.com (Y.H.H.); hhyun@khu.ac.kr (H.H.Y.)

**Keywords:** extended TPB, food waste reduction intentions, behaviors of wasting food, moderating effects, environmental literacy, consumer

## Abstract

This study applies an extended theory of planned behavior (TPB) to examine the determinants of consumers’ food waste reduction intentions, with subjective norm, attitude, perceived behavioral control, and guilt as key antecedents. In addition, this study investigates the moderating role of environmental literacy in strengthening these relationships. Using structural equation modeling, the proposed model demonstrated an acceptable overall fit, and most hypotheses were supported. The results reveal that attitude, perceived behavioral control, and guilt positively influence consumers’ food waste reduction intentions, whereas subjective norm does not exert a significant effect. Furthermore, food waste reduction intentions significantly reduce behaviors of wasting food. Regarding the moderating effects, environmental literacy strengthens the relationship between guilt and food waste reduction intentions, indicating that individuals with higher environmental literacy are more likely to translate moral emotions into behavioral intentions.

## 1. Introduction

As concerns about climate change grow and the movement toward environmental sustainability intensifies, food consumption and eating habits are becoming increasingly important [1]. Globally, one-third of the food produced for human consumption is lost or wasted, and reducing food waste at home, where environmental costs are highest, is believed to mitigate the negative impact on climate change [2]. While general waste is viewed as a necessary social evil to be disposed of and hidden, food waste is even more significant in that it is considered avoidable waste. It differs from general waste in that it is not ultimately considered waste but rather intended for disposal. Food waste is generally defined as food intended for human consumption that is ultimately discarded, lost, or uneaten [3]. This definition encompasses food that is removed from the human food supply chain at various stages, including production, distribution, and consumption. The amount of food wasted annually worldwide is estimated to be worth 440 trillion won (approximately $370 billion USD), equivalent to one-third of all food produced worldwide and enough to feed 800 million starving people. A recent report estimated that annual losses due to food waste will reach 670 trillion won (approximately $570 billion USD) by 2030, exceeding the annual gross national product of the welfare state of Sweden. This means that food waste equals the amount produced by a single nation. A major cause of food waste is consumer behavior. A significant amount of food is wasted due to consumer habits and ignorance, such as overpurchasing, overeating, consuming inconsiderately, not considering expiration dates, and improper storage methods [4]. For example, bulk purchases at large supermarkets or purchases through discount events often exceed consumers’ actual needs, leading to frequent discards of unused food before its expiration date [5]. Furthermore, a lack of planning when purchasing food and the habit of discarding leftover food are also identified as significant causes of food waste [2,6]. Therefore, a significant portion of food waste stems from intentional or unintentional consumer behavior [7]. To address the food waste problem, it is crucial to deeply understand consumer behavior and explore ways to change it. The need for this research is closely linked to the theory of planned behavior. Regarding food waste, the theory of planned behavior has been widely used as a theoretical lens to explain consumer behavior [8]. The theory of planned behavior provides a powerful theoretical framework for predicting and understanding consumer behavior. Consumers’ actions to reduce or increase food waste are determined by factors such as their attitudes (the belief that food should not be wasted), social pressure (the pressure from family or friends to reduce food waste), and their confidence in their ability to reduce food waste or feelings of guilt (the likelihood of planned food purchases and consumption) [9,10]. Therefore, for policies to reduce food waste to be effective, a deep understanding of why consumers waste food is necessary. This understanding, in turn, enables the development of strategies to change consumer behavior [1]. Furthermore, the issue of food waste is not simply a personal problem for consumers; it is an urgent societal issue that holds significant contemporary significance. Reducing environmental burdens and efficiently using resources are essential elements for creating a sustainable society [11]. Environmental literacy not only raises awareness of environmental issues but also plays a role in helping people perceive waste not merely as a burden to be discarded but as a valuable resource. It is believed that people with higher levels of environmental literacy are more likely to participate in waste value-added behaviors such as reducing food waste, reusing, and recycling.

Despite the increasing importance of food waste reduction, several significant research gaps still exist. First, existing studies applying the theory of planned behavior (TPB) have primarily focused on cognitive factors such as attitudes, subjective norms, and perceived behavioral control, while tending to overlook the role of moral emotions. In particular, guilt, a key driver of ethical consumption, has not received sufficient attention in the context of food waste reduction. Furthermore, while environmental literacy has been recognized as an important factor in eco-friendly behavior, the moderating effect of environmental literacy on shaping the relationship between emotion and behavior has not yet been sufficiently studied. To address these research gaps, this study aimed to expand the TPB framework by including guilt as a moral emotion and to analyze the moderating effect of environmental literacy on the relationship between the extended TPB and food waste reduction intentions.

## 2. Literature Review and Conceptual Model

### 2.1. Theory of Planned Behavior and Food Waste Reduction Intentions

The theory of planned behavior (TPB) has long been used as a behavioral model to predict and explain human behavior in various contexts [12]. TPB explains that behavioral intention (i.e., the will to act in a certain way) is the primary cause of behavior (i.e., the action taken). Specifically, the TPB posits that behavior is best explained through a person’s actual intention to exhibit that behavior. The TPB has been widely used to study human behavior toward the environment, including food waste behavior [13]. It has been proposed as a sound theoretical framework for examining food waste behavior in the household [11] and is utilized in available research on consumer perceptions and behaviors regarding food waste. Based on this theory, it can be argued that attitudes toward a behavior, perceived behavioral control, and subjective norms also influence the emergence of a behavior [14]. In other words, behavior can be considered a consequence of a specific intention because people anticipate the consequences of their actions, make decisions to achieve their chosen outcomes, and act accordingly [15]. Consumer attitudes, perceptions of control, and norms toward reducing food waste can influence their intentions to avoid food waste, which in turn influences their wasteful behaviors [16]. In a related study, Stancu et al. [17] found that behavioral control, shopping, and recycling behaviors are key determinants of food waste. They found that consumers who feel more negatively about food waste have a higher intention to reduce it, and that habits positively influence waste behaviors. Russell et al. [13] found that habits and emotions related to reducing food waste are the most important variables, while Filimonau et al. [18] found that lack of government support, incomplete laws, irresponsible consumer behavior, and limited internal resources contribute to food waste. Furthermore, Aydin and Yildirim [19] investigated the effects of knowledge about food preservation, moral attitudes, shopping, and eating habits on food waste behavior, finding that attitudes, knowledge, and habits significantly influenced food waste behavior.

Research related to a sub-variable of the TPB suggests that subjective norms represent the social pressure individuals perceive to perform a specific behavior. When people feel pressured to waste food, their food waste reduction intentions, i.e., to avoid creating food waste, increase [10]. Li et al. [1] also indicated that subjective norms positively influence food waste reduction intentions. Additionally, studies by Jia et al. [20] and Aydin and Aydin [11] reported that subjective norms significantly influence the intention to reduce food waste. When predicting consumers’ food waste reduction intentions, attitude refers to a favorable or unfavorable evaluation of an object. People feel bad when wasting food [17], worry when seeing wasted food [21], and feel guilty about throwing away food [22]. Therefore, it is expected that the belief that it is right to avoid creating food waste will positively influence intentions to avoid wasting food. Perceived behavioral control, expressed as the perceived difficulty of a behavior, is an individual’s perception of control over a specific behavior [12]. Also, as the severity or level of this control increases, individuals are able to perform a specific behavior more easily. According to the theory of planned behavior, when people perceive an appropriate level of control, their intention to engage in a specific behavior increases. From this perspective, an individual’s intention or behavior to avoid food waste is considered a situation within their control. Consequently, greater personal control can be associated with less waste or intention to waste [1]. Furthermore, studies such as Jia et al. [20] and Lin and Guan [23] found that perceived behavioral control significantly influenced intentions to generate food waste. Russel et al. [13] also suggested that PBC had the most decisive influence on intentions to reduce food waste. Furthermore, higher intentions to reduce food waste significantly influenced actual behaviors to reduce waste in daily life [9,24]. The application of the TPB as a framework for analyzing food waste generation behavior is often expanded by incorporating additional variables that influence human behavior [25]. The flexibility to expand TPB by adding various variables is one of its strengths [26]. The approach of including guilt in the TPB is often used in expanded models to supplement moral and emotional motivations that the existing TPB could not sufficiently explain [27]. The newly added variable, guilt, is a negative self-conscious emotion influenced by regret [28]. Consumers may experience this emotion due to behaviors that contribute to food waste. According to Kals et al. [29], emotional factors such as guilt play a significant role in ecological behavior. Most consumers experience guilt when engaging in wasteful behavior [11], and this discomfort can reflect moral dimensions and influence food waste behavior [30]. For this reason, individuals with a high level of moral guilt tend to avoid waste [31].

**Hypothesis** **1a.** *Subjective norms positively influence food waste reduction intentions.*

**Hypothesis** **1b.** *Attitudes positively influence food waste reduction intentions.*

**Hypothesis** **1c.** *Perceived behavior control positively influences food waste reduction intentions.*

**Hypothesis** **1d.** *Food waste reduction intentions positively influence food waste reduction intentions.*

**Hypothesis** **2.** *Food waste reduction intentions negatively influence food waste behavior.*

### 2.2. Moderating Effects of Environmental Literacy

**Figure 1:**
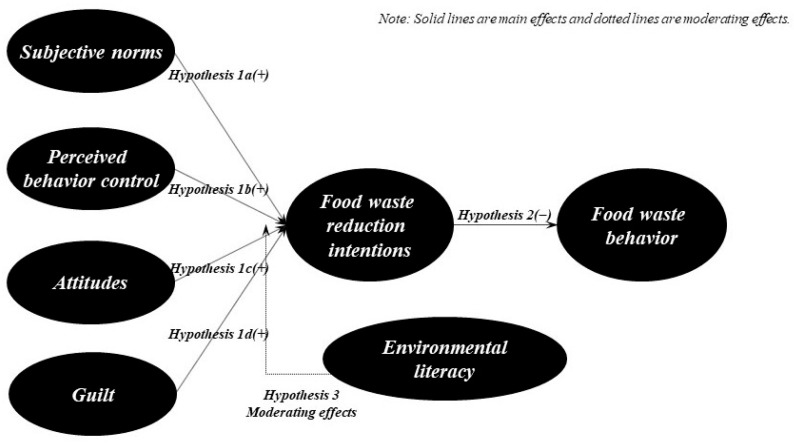
Research model.

Food waste reduction is a typical area of pro-environmental behavior where individual consumption and choices lead to environmental outcomes. Recent studies have examined the mechanisms by which intentions are formed, incorporating the theory of planned behavior. However, contextual discussions on the conditions under which this pathway operates more strongly have been relatively limited. In this regard, environmental literacy has been discussed as a key competency that helps individuals more accurately recognize the environmental consequences of their consumption and sustain pro-environmental choices [32]. While no studies have examined the moderating role of environmental literacy, similar studies include the following:

Purwanto et al. [33] suggested that environmental awareness positively influences intentions and behaviors related to food waste. Yildirim et al. [34] also found that higher environmental literacy strengthens environmental-related behaviors and decisions. Furthermore, Melnyk et al. [35] demonstrated that environmental awareness increases intentions to reduce food waste, suggesting that higher environmental competencies and knowledge lead to stronger connections between psychological drivers such as attitudes and intentions. Hu et al. [36] also reported that environmental literacy, which involves understanding the local environment, increases the expression of pro-environmental behaviors. Ghani et al. [37] indicated that environmental education positively influences sustainable food waste management behaviors, suggesting that it enhances cognitive and information acquisition abilities, thereby increasing environmental awareness and leading to pro-environmental behaviors. Furthermore, Kamalanon et al. [38] tested the moderating logic that environmental knowledge can strengthen the relationship between psychological antecedents and intentions in an extended TPB study targeting eco-friendly products. This suggests that environmental literacy can be utilized as a moderating variable to strengthen attitude- and norm-based intentions or behavioral pathways in the field of pro-environmental practices, although not specifically food waste. Furthermore, food waste is an environmental and ethical issue, and the more individuals are aware of this, the more readily they activate moral emotions. Also, the moderating role of environmental literacy is expected. Therefore, this study introduced environmental literacy as a moderator variable and hypothesized that the psychological pathways through which the extended TPB for food waste translates into reduction intentions would conditionally vary depending on environmental literacy levels. We hypothesized that higher environmental literacy would have a greater impact on the extended TPB and established the following hypotheses (Figure 1):

**Hypothesis** **3.** *Environmental literacy moderates the effects of the extended TPB on food waste reduction intentions.*

## 3. Methodology

### 3.1. Sample and Data Collection

For this study, samples were collected using non-probability sampling methods through a professional online panel provider. Participants were recruited from a large pool of panel members registered with the company. A pilot study was conducted one month prior to the main survey. This pilot study aimed to assess clarity, content, and response options; measure the time required to complete the questionnaire; and solicit feedback on additional suggestions and suggestions for improving the questionnaire. The pilot study led to the following steps: (1) deleting unclear questions and (2) revising questions that lacked clarity [39]. The response period was from 20 to 30 April 2025. Responses were obtained from respondents who gave voluntary consent, and respondents were notified in advance that their responses would be anonymized and that the collected data would be used solely for this study. A total of 600 questionnaires were distributed, of which 388 were used in the final analysis. Of the 600 collected responses, 212 were excluded due to incomplete or careless responses (e.g., linear responses, unusually short response times), and 388 valid responses were used for the final analysis. The sample size met the ratio of observations to estimated parameters (at least 10:1), ensuring sufficient statistical power and robustness of the parameter estimates. Respondents were 64.2% female and 35.8% male, with a balanced age distribution. Graduates of four-year universities or higher accounted for the largest proportion (60.6%). The average amount of food waste disposed of per session was 253 g (±231 g).

### 3.2. Instrument Development

The questionnaire for this study was prepared by back-translating the English questionnaire developed by Brislin [40] and Adler [41] into Korean. A native Korean researcher translated all questionnaire items into Korean, and an English expert then translated the Korean sentences back into English to identify any conceptual differences between the original and translated versions. The first section of the questionnaire, based on the extended theory of planned behavior [1], consisted of four factors and 16 items related to subjective norms, attitudes, perceived behavioral control, and guilt. The second section, the dependent variable, comprised three items regarding food waste reduction intentions; this subscale was based on the research of Stancu et al. [17]. Food waste behavior was measured by asking how much of the food purchased at home in a typical week is thrown away, categorized into fruits and vegetables, meat and fish, milk, and bread [31]. The measurement scale was measured as follows: hardly any (1), less than a tenth (less than 10%) (2), more than a tenth but less than a quarter (between 10% and 25%) (3), more than a quarter but less than a half (between 25% and 50%) (4), and more than a half (more than 50%) (5). The moderator variable, environmental literacy, consisted of four items based on the research of Kaya and Elster [32]. The final section measured demographic information, including gender, age, and education level. All items were rated on a 7-point Likert scale from 1 (strongly disagree) to 7 (strongly agree).

### 3.3. Statistical Analysis

Data analysis for this study was conducted using AMOS (Version 22, IBM, Armonk, NY, USA) and SPSS (Version 28, IBM, Armonk, NY, USA). Frequency analysis was used to identify demographic characteristics of respondents, and the validity and reliability of the measurement items were assessed through confirmatory factor analysis and reliability analysis [42]. To test the hypotheses, structural equation modeling and multi-group comparative analysis were conducted.

## 4. Results

### 4.1. Validity and Reliability Analysis

Before conducting a validity analysis, a Harmon test using exploratory factor analysis (EFA) was conducted to check for common method bias (CMB). The analysis revealed that the first factor’s explanatory power (33.9%) did not exceed 50% of the total explanatory power (75.1%). Therefore, the likelihood of CMB was deemed low, and the analysis was conducted accordingly.

Confirmatory factor analysis (CFA) was performed on all 22 measurement items (Table 1), resulting in seven factors (subjective norm, perceived behavior control, attitudes, guilt, food waste reduction intentions, behavior, and environmental literacy). The model fit was excellent (χ^2^ = 334.717; df = 188; *p* < 0.001; χ^2^/df = 1.780; GFI = 0.928; NFI = 0.954; TLI = 0.974; CFI = 0.979; IFI = 0.979; RMSEA = 0.045, RMR = 0.058), and the standardized coefficients for all measurement items were above 0.7. Furthermore, the average variance extracted was above 0.6, and the coefficient of correlation (CCR) and Cronbach’s alpha were both above 0.8, all of which were desirable. In addition, the square values of the correlation coefficients were all smaller than the AVE values, confirming the validity of the model [43,44] (Table 2).

### 4.2. Structural Equation Model

To test the hypotheses of this study, a structural equation model (SEM) was used (Table 3). The χ^2^ statistic indicated that the goodness of fit of the model in this study is at a relatively acceptable level (χ^2^ = 460.535; df = 124; *p* < 0.001; χ^2^/df = 3.714; GFI = 0.885; NFI = 0.926; CFI = 0.945; RMSEA = 0.084). The hypotheses of this study consisted of Hypothesis 1, which states that the extended TPB positively influences food waste reduction intentions; Hypothesis 2, which states that food waste reduction intentions positively influence consumer behavior; and Hypothesis 3, which verifies the moderating effect. When the extended TPB was applied to Hypothesis 1, perceived behavior control (β = 0.226; t = 5.841; *p* < 0.001), attitudes (β = 0.651; t = 10.714; *p* < 0.001), and guilt (β = 0.212; t = 5.574; *p* < 0.001) were found to have a positive effect on food waste reduction intentions, but subjective norms (β = 0.024; t = 0.016; *p* > 0.001) were found to have no significant effect, and this hypothesis was therefore partially accepted. These results are partially consistent with those of T’ing et al. [45] and Akhter et al. [46], who found that subjective norms do not effectively stimulate intentions to reduce food waste. This is because food waste disposal behavior is generally not observed by others, so there is no social pressure for individuals to engage in this behavior, and there are no strict social norms that inhibit such behavior [47]. Furthermore, the food waste reduction intentions were found to have a negative effect on the behavior of wasting food (β = −0.354; t = −6.456; *p* < 0.001), supporting Hypothesis 2. This result is consistent with previous studies that found that the higher the intention to reduce food waste, the more likely it is to lead to actual behavior in daily life [9,24].

Before analyzing the moderating effect of environmental literacy, we examined the measurement invariance of environmental literacy, grouping the groups based on their mean values, and confirmed that there were no issues. For each path in Hypothesis 3, models were compared using the χ^2^ difference, and significance was assessed considering both unrestricted and restricted degrees of freedom. The analysis results showed that guilt had a relatively stronger positive effect on food waste reduction intentions in groups with high environmental literacy, demonstrating a significant moderating effect (*p* < 0.05), leading to partial acceptance of Hypothesis 3 (Table 4). These results are partially consistent with previous research, which found that higher environmental literacy led to a stronger effect of guilt on food waste reduction intentions [33,35].

## 5. Discussion and Implications

### 5.1. Implications

The theoretical implications of this study are as follows: This study empirically validated the theoretical implications of an extended TPB model that incorporates guilt into the traditional TPB for explaining intentions and behaviors of not wasting food. This demonstrates that in moral and ethical consumption contexts, such as food waste, moral emotions, in addition to cognitive factors, can serve as key drivers.

Interestingly, subjective norms did not significantly influence food waste reduction intentions. This finding suggests that food waste reduction may be driven more by internalized moral values and perceived control than by external social pressure. This result is consistent with prior studies on pro-environmental behavior, which indicate that socially desirable behaviors such as waste reduction are often guided by personal norms rather than external expectations [48]. One possible explanation is that food waste is typically a private behavior, making it less susceptible to social evaluation. Therefore, this finding extends the TPB framework by suggesting that in moral consumption contexts, internal motivations may outweigh social normative influences. The significant effect of guilt on food waste reduction intentions highlights the importance of moral emotions in shaping environmentally responsible behavior. While the traditional TPB emphasizes cognitive determinants, this finding supports growing evidence that affective factors play a critical role in ethical consumption contexts. This result aligns with prior research suggesting that guilt can motivate reparative and prosocial behaviors [49]. In the context of food waste, guilt may arise from an awareness of environmental harm or moral responsibility, thereby strengthening behavioral intentions. The significant negative relationship between food waste reduction intentions and actual food waste behavior supports the fundamental assumption of TPB that intention is a direct antecedent of behavior. This finding reinforces the robustness of TPB in explaining environmentally relevant behaviors and confirms its applicability in the context of food waste. The moderating role of environmental literacy provides further insight into how cognitive and emotional processes interact. The finding that environmental literacy strengthens the relationship between guilt and intention suggests that knowledge enhances the salience and impact of moral emotions. This implies that individuals with higher environmental literacy are more likely to interpret food waste not merely as disposal but as a loss of valuable resources, aligning with the concept of waste valorization. In this sense, environmental literacy functions as a mechanism that transforms awareness into emotionally driven action. This finding contributes to the TPB literature by demonstrating that cognition and emotion are not independent but interact synergistically in shaping pro-environmental behavior. Visually presenting the environmental impact of wasted food and designing emotional messages that emphasize individual responsibility are also necessary. Rather than relying solely on information provision, interventions should target both cognitive and emotional dimensions of consumer behavior. Given that subjective norms were not significant, strategies focusing on social pressure may be less effective than those enhancing individual responsibility and perceived control. For example, more practical strategies might include providing education on food storage methods, providing guidelines for appropriate purchase amounts, and providing guidance on practices for reducing food waste. In addition, beyond improving behaviors aimed at reducing food waste generation, distinguishing between avoidable and unavoidable food waste is critical for developing effective waste management strategies. Accordingly, educational initiatives should be implemented to enhance individuals’ understanding of this distinction. Furthermore, the finding that environmental literacy enhances the influence of guilt suggests that environmental education should be implemented not simply as a knowledge transfer but as a foundation for guiding action. Consequently, schools, businesses, and public institutions should enhance environmental literacy through ongoing environmental education. Furthermore, developing and continuously exposing environmental content should enhance emotionally driven motivation for action. Furthermore, differentiated communication strategies should be designed based on environmental literacy levels. For groups with low environmental literacy, information- or education-focused strategies may be beneficial. Moreover, improving environmental literacy should go beyond knowledge transfer and emphasize the value creation potential of waste (e.g., composting and energy recovery), thereby encouraging consumers to perceive waste as a resource rather than a burden.

### 5.2. Limitations

Despite its theoretical and empirical contributions, this study is not without limitations. First, because the study was conducted exclusively on Korean consumers, the generalizability of the results is limited, and there are limitations to the cultural explanation. Furthermore, cross-national comparative studies are warranted, as environmental characteristics can vary depending on cultural and social norms. Second, although the extended TPB was used, its partial explanatory power remains limited because it did not include other moral extension variables besides guilt. Third, the cross-sectional and self-reported nature of the data limits causal inferences, and concerns about social desirability bias are raised, especially given the morally sensitive nature of food waste. Furthermore, because intentions, rather than actual behaviors, were measured, there may be a discrepancy between intended reduction behaviors and actual reduction behaviors. Third, although environmental literacy was modeled as a moderator variable in this study, its multidimensional structure may not have been fully captured, potentially weakening the observed interaction effects. Therefore, future research should consider a variety of individual differences, including general characteristics.

## 6. Conclusions

This study applied the extended theory of planned behavior to analyze factors influencing consumers’ food waste reduction intentions by categorizing them into four categories: subjective norm, attitude, perceived behavioral control, and guilt. The aim was to verify the moderating effect of environmental literacy. Structural equation modeling (SEBM) analysis revealed that the proposed model had an overall good fit, with most hypotheses supported. Specifically, among the extended TPB, attitude, perceived control, and guilt positively influenced food waste reduction intentions, while subjective norm had no significant effect. Furthermore, food waste reduction intentions negatively influenced the behavior of wasting food. It was found that the higher the consumers’ environmental literacy, the more positive the influence of guilt on the intention to reduce food waste, thus partially verifying the moderating effect.

## Figures and Tables

**Table 1:** The results of confirmatory factor analysis and reliability analysis.

Construct	Standardized Estimate	t-Value	Corrected Item–Total Correlation	CCR Cronbach’s Alpha
Subjective norm				0.898
SN_1_	0.913	fixed	0.646	0.927
SN_2_	0.874	25.526 ***	0.663	
SN_3_	0.912	27.848 ***	0.641	
Perceived behavior control				0.802
BC_1_	0.800	fixed	0.554	0.870
BC_2_	0.878	18.507 ***	0.612	
BC_3_	0.822	17.396 ***	0.655	
Attitudes				0.823
AT_1_	0.740	fixed	0.525	0.856
AT_2_	0.887	16.293 ***	0.526	
AT_3_	0.830	15.698 ***	0.533	
Guilt				0.906
GU_1_	0.888	fixed	0.670	0.942
GU_2_	0.936	29.094 ***	0.730	
GU_3_	0.937	29.134 ***	0.722	
Food waste reduction intentions				0.898
IT_1_	0.857	fixed	0.675	0.925
IT_2_	0.904	24.273 ***	0.664	
IT_3_	0.937	25.663 ***	0.680	
Behavior of wasting food				0.864
BE_1_	0.925	fixed	0.521	0.952
BE_2_	0.941	33.745 ***	0.516	
BE_3_	0.934	33.067 ***	0.539	
Environmental literacy as moderator				0.806
EL_1_	0.808	fixed	0.594	0.863
EL_2_	0.804	16.947 ***	0.573	
EL_3_	0.837	17.732 ***	0.592	
EL_4_	0.699	14.307 ***	0.557	

Note: CCR = composite construct reliability; *** *p* < 0.001.

**Table 2:** Correlation analysis and discriminant validity test results.

Construct	1	2	3	4	5	6	7	AVE	Mean ± SD
1. Subjective norms	1	*0.305*	*0.154*	*0.218*	*0.297*	*0.080*	*0.201*	0.809	5.52 ± 1.12
2. Perceived behavior control	0.552	1	*0.131*	*0.241*	*0.213*	*0.092*	*0.209*	0.695	5.14 ± 1.15
3. Attitudes	0.392	0.362	1	*0.162*	*0.212*	*0.075*	*0.168*	0.673	5.21 ± 1.02
4. Guilt	0.467	0.491	0.402	1	*0.205*	*0.085*	*0.320*	0.847	4.82 ± 1.23
5. Food waste reduction intentions	0.545	0.461	0.460	0.453	1	*0.122*	*0.193*	0.809	5.36 ± 1.11
6. Behavior of wasting food	0.282	0.304	0.273	0.292	0.349	1	*0.109*	0.870	4.38 ± 1.70
7. Environmental literacy	0.448	0.457	0.410	0.566	0.439	0.330	1	0.621	4.74 ± 1.05

Note: AVE = average variance extracted; all variables are significant at *p* < 0.01. Values in italics indicate squared correlations; SD = standard deviation. All items were measured on a 7-point Likert scale from 1—strongly disagree to 7—strongly agree.

**Table 3:** Structural estimate model.

Hypothesized Path (Stated as Alternative Hypothesis)	Standardized Coefficients	t-Value	Results
H1a(+) Subjective norm → Food waste reduction intentions	0.024	0.016	Not Supported
H2b(+) Perceived behavior control → Food waste reduction intentions	0.226	5.841 ***	Supported
H3c(+) Attitudes → Food waste reduction intentions	0.651	10.714 ***	Supported
H4d(+) Guilt → Food waste reduction intentions	0.212	5.574 ***	Supported
H2(+) Food waste reduction intentions → Behavior wasting food	−0.354	−6.456 ***	Supported
Goodness-of-fit statistics	χ^2^_(124)_ = 460.535 (*p* < 0.001) χ^2^/df = 3.714		
	GFI = 0.885 NFI = 0.926		
	CFI = 0.945		
	RMSEA = 0.084		

Note: *** *p* < 0.001.

**Table 4:** Moderating effects of consumers’ gender.

	High-EL (N = 166)		Low-EL (N = 131)		Unconstrained Model Chi-Square (df = 248)	Constrained Model Chi-Square (df = 249)	∆χ^2^ (df = 1)
	Standardized Coefficients	t-Value	Standardized Coefficients	t-Value			
H3a Subjective norm → Food waste reduction intentions	0.093	1.190	0.003	0.041	536.768	537.369	0.601
H3b Perceived behavior control → Food waste reduction intentions	0.238	3.215 **	0.324	4.173 ***		537.118	0.350
H3c Attitudes → Food waste reduction intentions	0.452	3.739 ***	0.510	5.772 ***		536.831	0.063
H3d Guilt → Food waste reduction intentions	0.255	3.043 **	0.154	1.801		541.240	4.472 *

Note: *** *p* < 0.001, ** *p* < 0.01, * *p* < 0.05.

## Data Availability

The data presented in this study are available upon request from the corresponding author.
